# Adaptive Cognitive Control in Prematurely Born Children: An HD-EEG Investigation

**DOI:** 10.3390/brainsci12081074

**Published:** 2022-08-13

**Authors:** Giovanni Mento, Lisa Toffoli, Letizia Della Longa, Teresa Farroni, Fiorella Del Popolo Cristaldi, Gian Marco Duma

**Affiliations:** 1 Department of General Psychology, University of Padova, 35131 Padova, Italy; 2 Padova Neuroscience Center (PNC), University of Padova, 35131 Padova, Italy; 3 Department of Developmental Psychology and Socialization, University of Padova, 35131 Padova, Italy; 4 IRCCS “La Nostra Famiglia-Medea”, Conegliano, 31015 Treviso, Italy; 5 Institut de Neurosciences des Systèmes, Aix-Marseille Université, 13005 Marseille, France

**Keywords:** premature birth, adaptive cognitive control, dynamic temporal prediction task, Wisconsin card sorting test, event-related potentials, contingent negative variation, brain source analysis

## Abstract

Preterm birth is a neurodevelopmental risk condition often associated with cognitive control (CC) impairment. Recent evidence showed that CC can be implicitly adapted through associative learning. In the present study we investigated the ability to flexibly adjust CC as a function of implicit stimulus-response temporal regularities in preterm (PT; N = 21; mean age 8 ± 1.3 years; gestational age 30 ± 18.5 weeks) and full-term (FT; N = 20; mean age 8 ± 1.3 years) school-age children. All children underwent an HD-EEG recording while undergoing the Dynamic Temporal Prediction (DTP) task, a simple S1–S2 detection task purposely designed to generate local-global temporal predictability of imperative stimuli. The Wisconsin card sorting test (WCST) was administered to measure explicit CC. The PT group showed more premature and slower (DTP) as well as perseverative (WCST) responses than the FT group. Moreover, pre-terms showed poor adaptive CC as revealed by less efficient global response-speed adjustment. This behavioral pattern was mirrored by a reduced and less sensitive to global manipulation anticipatory Contingent Negative Variation (CNV) and by different cortical source recruitment. These findings suggest that implicit CC may be a reliable endophenotypic marker of atypical cognitive development associated with preterm birth.

## 1. Introduction

It is estimated that between 30% and 40% of children born before term are at heightened risk of developing neurodevelopmental disorders, including autism spectrum disorders (ASD), attention deficit and hyperactivity disorders (ADHD), specific language impairment (SLI), learning disorders (LD) or clinically relevant problems in general [1]. Notably, even in the absence of diagnosed neurodevelopmental disorders, premature birth is statistically associated with multi-domain cognitive problems, involving sensory-perceptual, amnestic, verbal, visuospatial, learning and attentional-executive aspects [2]. These difficulties can often take a subclinical form that, nevertheless, may functionally limit the children’s proficiency not only in academic performance but also in social, relational and affective skills, ultimately impacting their quality of life [3]. Despite the large amount of work on the topic, it is still not clear which neurocognitive mechanisms may subtend an altered developmental trajectory in premature birth.

Most studies have traditionally focused on sensory and perceptual processing in the perinatal period or in the first months of life (see Mento & Bisiacchi, 2012 [4] for a review). However, in the last decade a lot of research has focused on tracing both neurobiological correlates and cognitive outcome associated with premature birth in the medium (childhood) and long (adulthood) term [5,6,7]. This developmental perspective is fundamental considering that several cognitive dysfunctions may be silent in the first years of life, but may emerge later, i.e., when environmental demands for controlled behaviour grow after the beginning of formal instruction in primary school. In particular, a disruption of cognitive control (CC) seems to be a core neuropsychological characteristic of prematurely born children [8]. CC, also known as executive function, is generally defined as the ability to regulate thoughts and emotions as a function of environmental demands, with the aim of overcoming automatism through volitional and goal-directed behaviour [9,10,11,12]. Due to the need to actively operate on sensory information to override automatic behaviour, CC has traditionally been considered a top-down function requiring an intentional effort to regulate behaviour based on explicit internal goals and external context [13,14]. However, a recent theoretical perspective supports a more ecological approach positing that CC can also be shaped by bottom-up processes, such as associative learning instantiated by both previous experience and contextual factors [15,16]. In this view, the ability to capitalize bottom-up resources becomes foundational for generating adaptive cognitive control (ACC).

Both simple associative and statistical learning have been advocated as two implicit mechanisms at the basis of ACC, as they allow the individual to extract and learn regularities from the surrounding environment since birth [17,18]. For example, a repeated stimulus-response (S–R) association can easily induce ACC, prompting participants to shift from a reactive to a proactive mode of control [19]. A repeated exposure to a biased environmental context also provides a rich source of predictive information that can be used to prepare for (and respond to) future stimuli in order to optimize cognitive processing at multiple levels, including visual perception [20,21], auditory perception [22], motor control [23,24,25], memory [26], interoception and emotion [27,28,29,30].

As a key point, whether acquired through implicit contextual information and/or prior experience, predictive knowledge can be proactively used as a proxy to up- or down-regulate attention and action preparation. This process aims to cope with upcoming events requiring controlled behaviour, for example speeding up expected responses or inhibiting prepotent but inappropriate ones, managing interference from distractors, or preparing for shifting tasks [31]. In other words, CC has been theoretically re-framed as an emergent property of prediction construction [32] rather than a supra-ordinate, static cognitive trait characterizing individual psychological profiles.

Crucially, while interest in ACC in healthy adults has generally increased in the last decade (see [16] for a review), the developmental evidence is still scarce. Yet a thorough understanding of ACC developmental trajectories would provide an exceptional opportunity to identify a fine-grained endophenotypic marker for all those risk conditions potentially impacting long-term neurodevelopmental outcome, including preterm birth. Some studies using contextual cueing paradigms showed that the encoding of stimulus regularities such as identity and location can guide selective attention in school-aged children [33,34], suggesting that CC can be easily driven by implicit contextual information encoded in memory. More recently, an emerging research line went a step further by proving that 5-year-old children succeed in engaging CC reactively in response to events, but they struggle to proactively prepare for them unless explicitly cued to use CC [35]. Instead, proactive CC seems to be spontaneously used from around 7 years of age [36,37,38]. In line with this, Gonthier and colleagues [39,40] investigated implicit CC in young children (5-year-old), older children (9-year-old) and adults by using a flanker task. Crucially, they manipulated the contextual information at both trial- (i.e., item-specific) and block-level (i.e., list-wide proportion congruency) in order to bias participants to expect mostly congruent or mostly incongruent trials. The results clearly showed that even preschoolers are able to learn to manage changing conflict levels and adjust their behaviour accordingly, without any explicit instructions to do so or awareness of it. Remarkably, they found no differences between older children and adults, showing that implicit CC stabilizes during childhood. By contrast, explicit CC knowingly continues to mature into young adulthood [10], suggesting a developmental dissociation between implicit and explicit CC. These findings aligned with a previous study of our laboratory [41], which used the Dynamic Temporal Prediction (DTP) task to investigate the ability of typically-developing children to adjust motor behaviour on the basis of either local (within-trial expectancy) or global (between-block expectancy) S–R temporal regularities. The findings clearly showed that, although in place at 5 years of age, the capability to implicitly and proactively adapt response speed as a function of global predictability becomes developmentally stable only after the age of 7, supporting the dissociation between explicit and implicit CC. 

Albeit limited, the extant evidence on typical development suggested the possibility of assessing ACC as a marker of potential clinical relevance for atypical development, which has been scarcely investigated. In two studies using the DTP task we were able to show that the ability to implement ACC on the basis of global predictability is disrupted in Down syndrome [42] and focal epilepsy of childhood [43]. Conversely, deaf children with cochlear implants (a condition of neurodevelopmental risk for academic outcomes; [44]) showed comparable ACC performance as typically hearing children, suggesting that implicit learning might be resilient to early adverse experiences such as a lack of auditory stimulation [45]. This encourages investigation of the mechanisms underlying ACC in populations presenting a condition of major risk for neuropsychological development and academic outcome, such as the case of preterm birth. Yet, as far as we know, it is still an unaddressed question as to whether ACC is impaired in preterm children.

In the present study, we used the DTP and the Wisconsin Card Sorting Test (WCST; [46]) as two sensitive tools to respectively investigate implicit and explicit ACC in a sample of preterm (PT) school-aged children compared to a full-term (FT) age-matched control group. Noteworthy, unlike traditional cognitive control tasks (i.e., Stroop, flanker or Simon) the DTP task does not include a conflict condition, ruling out any potential confound deriving from excessive working memory load or complex instructions, as the only instruction is to press a button when the target occurs. This makes it suitable for investigating ACC in young typically [41] and atypically [42,43,45] developing children, including PT children. Nonetheless, in the context of the DTP, task ACC is crucial for the efficient adaptation to both local and global patterns. Indeed, optimal behavioral adaptation should reflect not only the generation of adequate predictive models of events’ sensory structure, but also optimal flexibility in updating and implementing these predictions to proactively prepare for action by balancing excitatory and inhibitory neural mechanisms. To unravel the neural activity underpinning this cognitive function, the high spatial resolution electroencephalographic activity (HD-EEG) was recorded and compared between-groups while participants were performing the DTP. 

In line with previous literature [8] the general hypothesis of the study was to observe a less efficient CC in PT than FT children both at the behavioral and neurofunctional level. More specifically, as a first hypothesis (H1), we expected the PT group to show an overall poorer behavioral performance than the FT group, with slower, more premature responses in the DTP task (implicit CC), as well as more perseverative errors in the WCST (explicit CC). As a second hypothesis (H2), we expected the DTP task to succeed in inducing ACC in all children. Specifically, we expected behavioral responses to conform to both trial-wise (local) and block-wise (global) stimulus predictability, respectively [23,41,43,47]. We also hypothesized (H3) between-group differences only in the ability to use global, rather than local, predictability to adjust behaviour. Specifically, in line with previous evidence showing suboptimal global ACC in children with atypical development (Down Syndrome, [42] and suffering neurological disorders [43], we expect PT children to show a less efficient global behavioral adaptation than FT peers even in the absence of diagnosed neurodevelopmental disorders. Finally, in line with our previous findings [23,25], we hypothesized (H4) that the group-level behavioral differences would be supported by a less efficient neural engagement in PT children. Specifically, we targeted both pre-stimulus (Contingent Negative Variation or CNV) and post-stimulus (P1, N1, P2, N2, P3) ERP components as specific electrophysiological markers sensitive to expectancy/response preparation and stimulus processing/response implementation, respectively. The scalp-level analyses were complemented with a source-level exploration, with the aim of unraveling the spatiotemporal neuro-dynamics of distinct neurocomputational stages underlying CC. 

## 2. Materials and Methods

### 2.1. Participants

Data were collected from 21 prematurely born children (PT group; mean age = 8.4 ± 1.3 years, range = 6–11 years; 7 boys; 16 right-handed) and 21 age- and gender-matched children born at term (FT group; mean age = 8.2 ± 1.4 years, range = 6–11; 8 boys; 16 right-handed). Two children were excluded from the PT group and one from the FT group due to excessive noise during EEG recording. Therefore, the final sample was N = 39 (19 PT and 20 FT; see Table 1 for the demographic characteristics). PT participants were recruited from the association ‘‘Pulcino’’ in Padua, a center that provides support for premature infants and their families from the earliest stages of development since later childhood. Participants in the FT group were recruited from the local community. Visual acuity was normal or corrected to normal. Inclusion criteria in the PT group were birth before 35 gestational weeks and acceptance of the study protocol by the parents. Exclusion criteria were major risk conditions for neurocognitive development, including craniofacial malformations, genetic syndromes, major neurological diseases, ultrasound evidence of severe intra-ventricular hemorrhage at birth or during the permanence in the Neonatal Intensive Care Unit (III–IV grade by Volpe, 2000), abnormal brainstem auditory-evoked potentials (BAEPs) at birth, or diagnosis of neuropsychiatric or neurodevelopmental disorders at testing time. Participants’ characteristics are summarized in Table 1. All children’s parents signed a written consent form. All experimental procedures were approved by the Ethics Committee of the School of Psychology at the University of Padua (protocol n° 2536) and were conducted according to the principles expressed in the Declaration of Helsinki.

### 2.2. Experimental Procedures

All families were invited to join the interdepartmental University’s HD-EEG laboratory. All participants’ parents signed a written informed consent. After familiarizing themselves with the HD-EEG lab setting, children comfortably sat down in front of a monitor and the HD-EEG net was applied. Each child undertook a six-minute resting state recording and then started the Dynamic Temporal Prediction (DTP) task [41]. The DTP is a warned, simple response time (RT) task purposely designed to investigate how behavioral performance and neurophysiological activity are implicitly shaped by stimulus occurrence probability over time. In this task, the way individuals prepare to respond to the imperative stimulus depends on both local and global prediction. For local prediction we mean how long participants wait for the imperative stimulus within each single trial (hazard rate), whereas for global prediction we mean how likely is the imperative stimulus occurrence after a short or long preparatory interval on the basis of contextual information. Specifically, as detailed below, the proportion of short, medium and long stimulus onset asynchrony (SOA) intervals between the warning and the imperative stimulus is manipulated list-wide and covertly. The manipulation generates “fast” (short-expectancy biased) or “slow” (long-expectancy biased) experimental blocks with respect to a “medium” speed baseline condition (uniform distribution). In this way it is possible to test the participants’ ability to adjust their motor control (speed response) on the basis of bottom-up implicit task demand. The DTP task has proven to be useful at unraveling the neural bases [23] as well as at tracking both typical [41] and atypical [42,45] developmental trajectories of ACC. 

After completing the experimental task, all participants underwent a cognitive assessment as a part of a wider research project. Non-verbal intelligence was measured through the Raven’s Colored Progressive Matrices (CPM) test [48]. The raw score obtained through the Raven’s CPM test was used to exclude participants whose mental age was below 2 standard deviations (SD), in consideration of their age average. For this purpose, the Italian normative data [49] were used. Explicit cognitive flexibility was tested by using a computer-based version of the Wisconsin Card Sorting Test (WCST) [46]. Specifically, we used a custom computerized version with a rule-switch every 10 trials for a total of 128 cards.

### 2.3. Experimental Task

**Figure 1:**
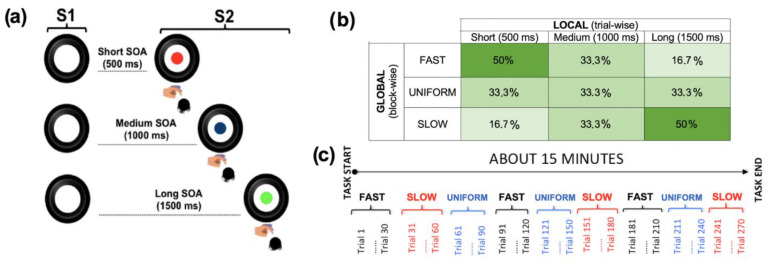
Representation of the DTP task. (**a**) Trial structure. The circle (S1) warned children on the forthcoming presentation of the imperative S2 stimulus (an animal cartoon; here represented with colored disks for illustrative purposes). Participants had to make speeded reaction times at S2 onset by pressing the space button on the keyboard. The effect of local prediction was assessed by manipulating S1–S2 stimulus onset asynchrony (SOA) within each experimental block, generating three fixed intervals of 500 ms, 1000 ms and 1500 ms. (**b**) Experimental Design. The effect of global prediction was assessed by manipulating the between-block a priori percentage of each SOA to create three probabilistic distributions in which the SOAs were skewed toward the short (fast block), equally distributed (uniform block) or long (slow block) SOA. (**c**) Task design. A total of 9 blocks were delivered. Each block included 30 trials, for a total of 270 trials. The block order was randomized between-subjects. The picture shows an example of possible order randomization.

We used a laptop with a 17-inch monitor at a resolution of 1280 × 1024 pixels to present the stimuli. Participants were seated comfortably in a chair at a viewing distance of around 60 cm from the monitor. The trial structure is shown in Figure 1. In each trial the first event was the display of a warning visual stimulus (S1), which was followed by an imperative visual stimulus (S2) lasting on the screen for a maximum of 3000 ms. S1 consisted of a picture of a black camera lens surrounded by a circle (total size of the stimulus: 840 × 840 pixels, 144 dpi, 10.62° × 10.54° of visual angle). S2 was a picture of a cartoon character, displayed centrally within the camera lens. The inter-trial-interval was random between 600 and 1500 ms. Participants were asked to make speeded target detection by pressing the spacebar with the index finger of the dominant hand as quickly as possible at target onset.

#### 2.3.1. Local Predictive Context

The local stimulus predictability was investigated by manipulating the S1–S2 SOA trial by trial within each experimental block, leading to three possible fixes. These included a short (500 ms), a medium (1000 ms), or a long (1500 ms) SOA (Figure 1a). In this way we were able to generate in each block three levels of local temporal preparation to S2 onset. This in turn is expected to change the subjective temporal expectancy of target onset [50,51,52,53,54,55]. Specifically, in line with previous literature (see [51] for a review), we expected participants to be fastest at detecting the targets occurring at the longest SOA and slowest at those appearing at the shortest SOA.

#### 2.3.2. Global Predictive Context

**Figure 2:**
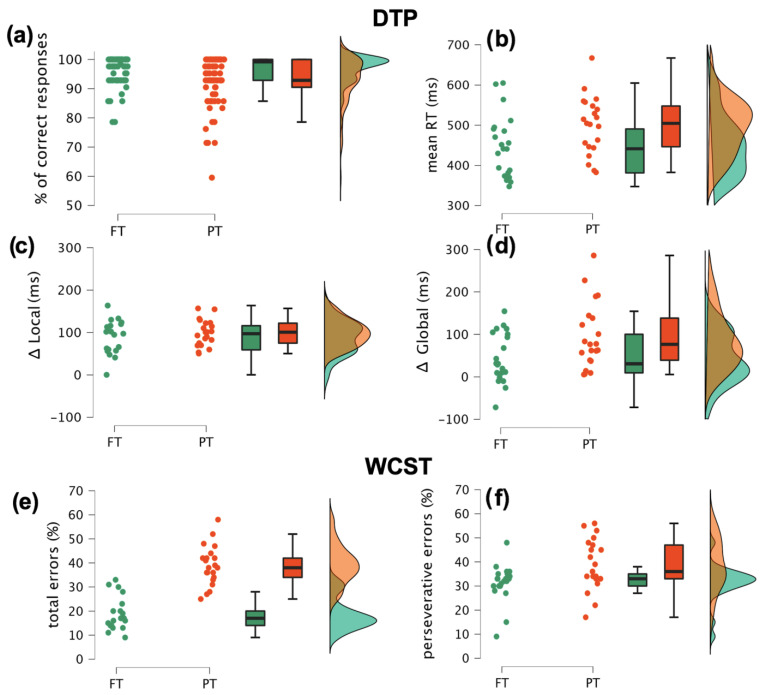
Behavioral data. The figure shows the single-subject values (dots), the box plots and the rainclouds clustered by group. The four upper panels refer to the DTP task and represent the global mean response accuracy (**a**), the global mean response speed (**b**), the mean response speed adjustment as a function of local (Δ local = short SOA RTs minus long SOA RTs) (**c**) and global (Δ global = slow block RTs minus fast block RTs) (**d**) stimulus predictability. The two bottom panels display the percentage of total (**e**) and perseverative (**f**) errors at the WCST task.

To investigate the global predictability, different frequency distributions per each SOA length were introduced and manipulated list-wide in order to create three different block types. See also Figure 2 for a description.

##### Uniform (U) Blocks

In the uniform block the three SOA intervals had the same probability of occurring (33,3% each). This type of distribution has been traditionally employed in both adult [51,56,57,58] and developmental [25,57,59,60] literature and is known to translate into a biased a posteriori temporal preparation. Indeed, as time elapses, the conditional probability of S2 onset increases exponentially since it has not occurred yet [47]. Hence, motor preparedness will be lowest at the shortest SOA and highest at the longest SOA.

##### Fast Blocks (Short-Biased)

In this case, most of the SOA intervals were short. In particular, the relative frequency distribution was 50%, 33.33%, and 16.67% for the short, medium, and long SOA, respectively. This manipulation turned out into a speeding up of the global task speed, as the stimuli were mostly interspersed with short waiting intervals.

##### Slow Blocks (Long-Biased)

In this block we used an aging distribution [47,61], which is known to enhance the hazard-based increment of temporal expectancy as a function of SOA length. Specifically, the relative percentage was 16.7%, 33.3%, and 50% for the short, medium, and long SOA, respectively. In this case we obtained a general slowing down of the stimulus presentation rate as most of the waiting intervals between stimuli were long.

### 2.4. Experimental Design

The experimental design is shown in Figure 2. The manipulations yielded a factorial design with SOA length (short vs. medium vs. long) and block type (fast vs. uniform vs. slow) as two factors orthogonally contrasted to investigate how behavioral performance is independently affected by local and global stimulus predictability.

For each block we delivered 30 trials and each block was presented three times, leading to nine experimental blocks and 270 trials. Specifically, we delivered a total of 45, 30 and 15 trials respectively for short, medium and long SOA in the fast block; 30 trials for each SOA in the Uniform block; and 15, 30 and 45 trials respectively for short, medium and long SOA in the slow block. Each block required the same sensorimotor engagement, as the visual stimuli and the motor response were always the same regardless of SOA length or block type. The experiment lasted about 15 min. It is important to notice that participants were not aware of either local or global manipulations since these parameters were not made explicit by the researchers. Furthermore, the blocks were delivered continuously. Resting pauses were blank slides inserted at the middle of each block in order to avoid cueing participants about between-block shifts. The block-type order was randomly sorted between subjects in order to avoid any potential implicit predictive bias due to fixed SOA or block-type order. After completing the task all participants were asked if they had become aware about any change in speed across the task. They only reported a general change in local stimulus predictability (i.e., that they could wait shorter or longer to see the target) but did not realize any specific global pattern. A block of 20 training trials for each condition was delivered before the experimental session to ensure if they understood task instructions. In this practice block, a yellow smile with a neutral expression was displayed when either premature (<150 ms from target onset) responses were provided. A yellow smile with a happy expression was displayed if the RT was between 1000 and 1500 ms from target onset. Finally, a green smile with a happy expression was displayed if the RT was between 150 and 1000 ms. E-prime 2 software (Psychology Software Tools, Pittsburgh, PA, USA) was used to create and administer the stimuli. 

### 2.5. Behavioural Data Analysis

Both mean accuracy (percentage of not premature responses) and RTs to targets were collected and analyzed separately for each group. Specifically, in order to obtain a general measure of the ability to accomplish the task, the mean number of errors (i.e., premature responses < 150 ms from S2 onset) across all experimental conditions was calculated for each participant. All children showed high accuracy (>90% of corrected responses) and were included in the analyses. With regard to RTs, only responses between 150 ms and 1500 ms from target onset were considered correct and included in the analyses. We fitted separate analyses of variance (ANOVA) on both the DTP (mean error percentage and RTs) and the WCST. In the latter case we considered two measures, i.e., the mean percentage of perseverative response (i.e., when the participant continues with the same response strategy following a rule switch) and of total error (including both perseverative error, and non-perseverative/random error).

We considered the group (PT vs. FT) as a between-subject factor. To reduce the number of multiple comparisons, only the two extreme SOA intervals (short vs. long) and block-type (fast vs. slow) were entered in the GLMs as within-subject factors indicating local and global prediction, respectively. This choice was also justified by previous findings showing that in the DTP task these conditions yielded the maximum experimental effects on RTs [41]. Chronological age was also added as a covariate to partial out any potential maturational age effects, given the wide age range included in the study. To directly compare the effect of different hierarchical levels of predictability on task performance, we also calculated the delta (Δ) scores as the mean RT difference between short and long SOA (Δ local prediction) and between fast and slow blocks (Δ global prediction). More specifically, the Δ global index was calculated only for short-SOA trials as previous studies clearly showed that behavioral adaptation in terms of response speed is negligible for long-SOA trials, due to the hazard-function [24,53,54,56].

Both the individual Δ DTP values and the mean WCST scores (i.e., the individual mean percentage of both total and perseverative errors) were entered in a univariate ANOVA to test for between-group effects. Finally, we applied partial, age-mediated Pearson’s correlations among demographic variables (i.e., both gestational age and birthweight) and behavioral scores, including the individual local and global Δ prediction indexes in the DTP task and both the total and perseverative individual percentage of errors in the WCST. This helped us to better unravel the relationship between maturational factors on the one side and implicit-explicit CC on the other side.

### 2.6. EEG Recording and Preprocessing

The high-spatial resolution EEG signal was recorded trough a 128-channel Geodesic high-density EEG System (EGI^®^ GES-300), with electrical reference to the vertex. We used a sampling rate of 500 Hz and kept the impedance below 60 kΩ for each electrode. Signal preprocessing was performed through EEGLAB 14.1.2b [62]. The continuous EEG signal was first down-sampled at 250 Hz and then bandpass-filtered (0.1–45 Hz) using a Hamming windowed sync finite impulse response filter. After filtering, the continuous signal was epoched between 500 and 1996 ms from S1 onset and between −100 and 900 ms from S2 onset to target pre- and post-stimulus windows, respectively. Epochs related to trials containing premature responses were rejected. Epoched data were entered to an automated bad-channel and artifact detection algorithm including the TBT plugin implemented in EEGLAB. This algorithm identified the channels exceeding a differential average amplitude of ±250 μV. These channels are then marked for rejection. Channels that were marked as bad on more than 30% of all epochs were excluded. Epochs including more than 10 bad channels were also excluded. Data cleaning was performed by means of an independent component analysis [63], using the Infomax algorithm [64] implemented in EEGLAB. The resulting independent components were visually inspected in topography and time-series, and those clearly related to eye blinks, eye movements, muscle artifacts and heartbeat were discarded. The remaining components were then projected back to the electrode space to obtain cleaner EEG epochs. Finally, bad channels were reconstructed with the spherical spline interpolation method [65]. The data were then re-referenced to the average of all electrodes, and baseline correction was applied by subtracting the mean signal amplitude in the pre-stimulus interval. Epoched data were imported in Brainstorm [66] to generate the individual average for each electrode site and experimental condition. We applied a weighted average in order to control for the unbalanced number of epochs per condition [67]. 

### 2.7. EEG Statistical Analysis

We applied a whole-scalp analysis approach at all electrode sites using a paired t-test (α < 0.05) permutation approach to control the family-wise error rate [68]. To control for the 1-type error we performed 2000 Monte-Carlo permutations and applied cluster-based correction over all 128 electrode locations using the Fieldtrip functions [69], accessible via Brainstorm. 

The ERP analysis focused on specific ERP components that proved to be sensitive markers of the different cognitive mechanisms underlying implicit cognitive control [23,25]. Specifically, to investigate the expectancy implementation stage, which consists in the ability to prepare for detecting targets, the late Contingent Negative Variation (CNV; [70,71]) was targeted and measured during response preparation (pre-stimulus interval). Namely, we considered the mean voltage amplitude over a 200-ms period immediately before S2 onset. This extended over a window of 300 to 500 and 1300 to 1500 ms from S1 onset for short and long SOA trials, respectively. To investigate the response implementation stage, which consists in the ability to translate temporal expectancy into optimized stimulus processing and motor responses, the main post-stimulus ERP components were targeted and measured. Namely, in line with both previous studies (Mento and Vallesi, 2016) and with the grand average visual inspection, we targeted the P1 (80–140 ms), N1 (140–200 ms), P2 (200–300 ms), N2 (300–400 ms) and P3 (300–500 ms) components. 

To investigate between-group differences in CC we applied a first-level analysis by collapsing all block-type conditions and comparing the neural activity in FT vs. PT children, separately by SOA length. In this way we were able to directly compare both pre- and post-stimulus ERP activity for different levels of local predictability, regardless of the effect of block-wise global changes in stimulus temporal predictability. Then, to specifically investigate ACC we run a second-level, within-group analysis by comparing the neural activity in fast vs. slow blocks separately by SOA (short vs. long). In this way we directly addressed how the neural pre- and post-stimulus activity was shaped by global predictability within each group. 

### 2.8. Cortical Source Modelling

To investigate the neural generators underlying both expectancy and response implementation we applied a cortical source estimation. To this purpose baseline-corrected epochs were imported in Brainstorm [66] to model their cortical generators. We used the ICBM152 anatomical template to approximate the individual anatomy of each participant [72]. Co-registration of EEG electrodes position was performed via Brainstorm, by projecting the digitized EEG sensor positions GSN Hydrocel 128 E1 available in Brainstorm on the head surface. We then derived an EEG forward model using the three-layer boundary element method (BEM) from OpenMEEG implemented as a Brainstorm routine [73,74]. The source space was constrained to the cortex and modeled as a grid of 15.002 orthogonal current dipole triplets. We used sLORETA as a source model, with Brainstorm’s default parameter settings. The empirical noise covariance model was obtained from the average of ERP baseline signals. The sources were projected to the standard anatomical template (MNI) and their activity was transformed in Z scores relative to the baseline. Finally, a spatial smooth with a FWHM of 3 mm was applied to each source. The cortical activations were located according to the anatomical Desikan-Killiany atlas [75] adapted for cortical space solution.

## 3. Results

### 3.1. Behavioural Results

#### 3.1.1. Dynamic Temporal Prediction Task

The mean accuracy scores and RTs per group and condition are reported in Table 2 and Table 3, respectively. Overall, accuracy scores were very high with all children showing on average correct (i.e., not premature) responses in more than 95% of trials. The ANOVA revealed a significant group effect (F(1, 39) = 6.9; *p* < 0.02; η^2^p = 0.15) with PT children committing more premature responses (93.3 ± 5.2%) than FT children (96.9 ± 2.53%). As expected, the mean accuracy was also dependent on age (F(1, 39) = 6.56; *p* < 0.02; η^2^p = 0.14), as the percentage of premature responses decreased with increasing age. No other main or interaction effects were found.

The behavioral benefits conferred by the experimental manipulation were observed mainly in response times (RTs; Table 3). The ANOVA revealed a significant group difference (F(1, 39) = 4.14; *p* < 0.02; η^2^p = 0.1), with PT children showing on average slower responses (499 ± 77 ms) than FTs (448 ± 72 ms). We also found an expected main effect of age (F(1, 39) = 3.87; *p* < 0.05 η^2^p = 0.09), as RTs speeded up with increasing age. Moreover, the ANOVA revealed a main within-subject effect, as the response speed significantly depended on trial-type (SOA effect: F(1, 39) = 12.22; *p* < 0.01; η^2^p = 0.24). Specifically, all children were faster at detecting targets when these were locally preceded by long as compared to short SOA preparatory intervals (mean speed advantage = 93 ms; t = 17.1; *p* < 0.01; Cohen’s d = 1.12), although this effect interacted neither with group nor age (all Fs(1,39) < 1; all ps > 0.36; all η^2^p_s_ < 0.01). In other words, both FT and PT children were comparably good at exploiting the local temporal predictability to generate temporal prediction and consequently proactively adapt CC to speed up their responses. 

Noteworthy, we found a significant block-type × group interaction (F(1, 39) = 3.78; *p* < 0.05; η^2^p = 0.9). From the post-hoc tests it emerged that, while all children showed overall faster RTs in fast than slow blocks (mean difference = −48 ms; t = −5.16; *p* < 0.01; Cohen’s d = −59), this advantage was relatively larger in PT (mean speed advantage = −65 ms; t = −5.33; *p* < 0.001; Cohen’s d = 0.79) as compared to FT children (mean speed advantage = −31 ms; t = −2.58; *p* < 0.05; Cohen’s d = −38). This interaction was further addressed by directly comparing between-group the Δ local and global prediction indexes (calculated only for short-SOA trials) as two behavioral markers reflecting different hierarchical levels of adaptive cognitive control. The univariate ANOVAs revealed significantly larger global (F(1, 40) = 5.7; *p* < 0.03; η^2^p = 0.12) but not local (F(1, 40) = 1; *p* < 0.32; η^2^p = 0.02) Δ values in PT than FT children. The single-subject data are shown in Figure 2c,d.

#### 3.1.2. Wisconsin Card Sorting Test

The univariate ANOVA revealed a significant between-group difference in the WCST performance. Specifically, PT children reported a higher percentage of both total errors (F(1, 40) = 76.4; *p* < 0.001; η^2^p = 0.66) and perseverative responses (F(1, 40) = 29.4; *p* < 0.001; η^2^p = 0.42) than FT children, as shown in Figure 2e and Figure 2f, respectively.

#### 3.1.3. Correlational Analyses

**Figure 3:**
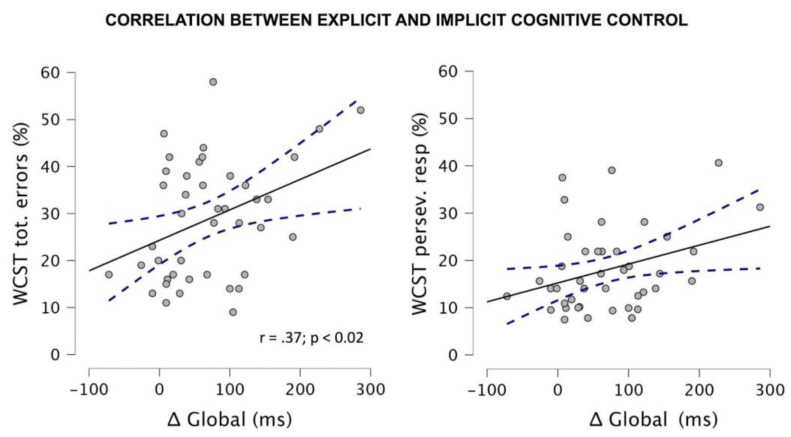
Correlational results. Correlation between Δ Global single-subject values at the DTP task and the percentage of total errors (left panel) and perseverative errors (right panel) at the WCST task.

We also found that in the PT group the gestational age was inversely correlated with the percentage of total errors in the WCST (r = −0.47; *p* < 0.05), with more premature children committing more errors. Yet, no significant correlations between maturational variables (gestational and birthweight) and DTP adaptive local or global Δ indexes emerged (all rs = 0.12; all ps > 0.6). Most interestingly, we found that the Δ global index correlated with both the individual percentage of total (r = 0.37; *p* < 0.02) and of perseverative errors (r = 0.32; *p* < 0.05) in the WCST. By contrast, no significant correlations were found for the Δ local index and the WCST scores (r < 0.22; *p* > 0.1). These findings are shown in Figure 3 and suggest that explicit and implicit CC are moderately related.

### 3.2. EEG Results

#### 3.2.1. Between-Group Comparison

**Figure 4:**
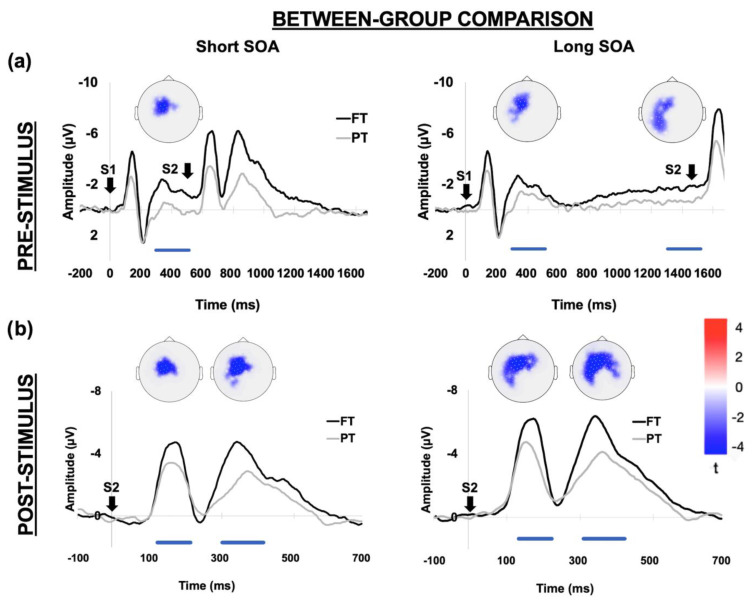
Between-group ERP comparison. The upper panel (**a**) represents the statistically significant electrodes exceeding the critical t-score threshold for statistical significance over the pre-stimulus window (stimulus expectancy/response preparation). The negative cluster indicates that ERP mean amplitude is significantly larger (i.e., more negative) in the FT than in the PT group for both short and long SOA intervals over left-central electrodes. These effects extended over a 300 ms window before S2 onset, including a N2/CNV modulation for the short SOA condition and a CNV modulation for the long SOA condition. The ERP waveforms below the scalp maps show the time series derived by averaging the electrodes included in the negative cluster. The S1 at 0 ms indicates the ERP time locking, while S2 indicates the onset of the imperative stimulus. The lower panel (**b**) represents the same analysis performed over the post-stimulus window (stimulus processing/response implementation). The negative cluster indicates that ERP mean amplitude is significantly larger (i.e., more negative) in the FT than in the PT group for both short and long SOA intervals over left-central electrodes in two distinct windows, covering the N1 and N2 temporal range. The ERP waveforms below the scalp maps show the time series derived by averaging the electrodes included in the negative cluster. The S2 at 0 ms indicates the ERP time locking. For all comparisons the colored horizontal bars represent the timing of significant ERP effects.

As shown in Figure 4 the visual inspection of the grand-averaged ERP waveforms clearly shows larger pre- and post-stimulus amplitude voltages in FT as compared to PT children, suggesting greater recruitment of neural resources during both stimulus expectancy and response implementation stages. This effect was confirmed by the first-level statistical analysis aimed at identifying group-level differences after collapsing block-types. Indeed, the permutation analyses in the pre-stimulus time window identified a left-central cluster of electrodes which consistently showed more negative activity in FT children for all SOA conditions. More specifically, in the short-SOA trials we identified an early effect in the window between 300 and 500 ms from S1 onset (*p* < 0.02, cluster statistic (c) = −56, cluster size (s) = 38), which presumably incorporated both S1-locked (N2) and anticipatory (CNV) activity (Figure 4a). Indeed, due to the narrow preparatory window it was not possible in this case to temporally disentangle these two computational stages in a reliable way. This hypothesis is further confirmed by the fact that in long-SOA trials, which allow for a longer motor preparation, we still found an early effect between 300 and 500 ms (FT vs. PT *p* < 0.02, cluster statistic (c) = −59, cluster size (s) = 44), which was nevertheless followed by a much later CNV modulation between 1300 and 1500 ms (*p* < 0.02, cluster statistic (c) = −61, cluster size (s) = 50). Noteworthy, regardless of their timing, all these effects were spatially localized over a cluster of left-central electrodes, in line with the traditional CNV scalp distribution [70,71]. Nonetheless, this cluster extended over more posterior sites in long-SOA trials, suggesting at least partially different neural generators on the basis of the available preparatory interval. When applied in the post-stimulus window the permutative analysis also yielded a larger negativity in the FT group both in the N1 (140–200 ms) and N2 (200–300 ms) range. As observed for the CNV, these effects were localized over a left-central cluster of electrodes and extended over posterior areas for long SOA trials (Figure 4b). 

**Figure 5:**
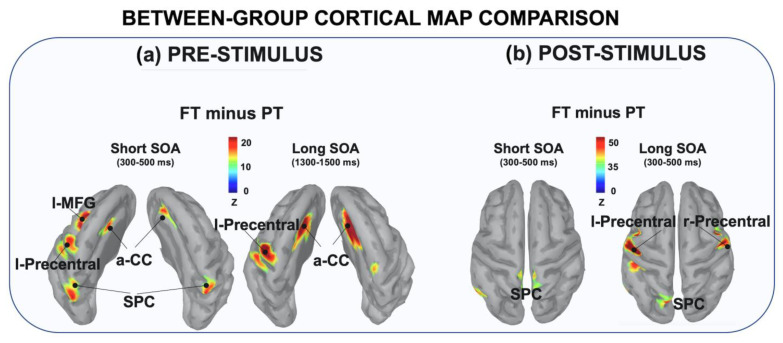
Between-group differential cortical map reconstruction. The pictures display the differential (FT minus PT) cortical map reconstruction relative to each significant ERP effect for pre-stimulus (**a**) and post-stimulus (**b**) temporal windows. Data were adjusted using a threshold of 50% of the maximum amplitude and a size of at least 10 vertices and they were reported on a template cortex smoothed at 40%.

The cortical source reconstruction applied over the pre-stimulus ERP window showed that during the stimulus expectancy stage FT children recruited a distributed frontal cortical network including the left precentral (l-Precentral), left middle-frontal gyrus (l-MFG) and the bilateral anterior cingulate cortex (a-CC). In addition, only in short-SOA trials did this network extend further posteriorly, involving the superior parietal cortex (SPC) areas bilaterally. By contrast, PT children showed a lower degree of cortical activity, which was mainly focused over the SPC with a very small activity extending over frontal cortical regions. This different pattern of cortical activity during the pre-stimulus window is clearly shown in Figure 5a, which depicts the differential activity obtained by subtracting the cortical reconstructed maps (i.e., FT minus PT). Specifically, for both short and long SOA conditions the FT group showed a greater recruitment of neural resources over the bilateral a-CC and the l-Precentral gyrus. In addition, only in short SOA trials was an additional fronto-parietal involvement found, including the l-MFG and the bilateral SPC. As observed for the pre-stimulus window, the reconstructed maps obtained from the post-stimulus ERP activity also showed higher cortical activity in FT than PT children over bilateral occipital, temporal and parietal areas. The differential map relative to the N2 range (300 to 500 ms from S2 onset; Figure 5b) further narrowed this differential pattern over the parietal areas (SPC) for both short and long SOA trials. In the latter case we also observed a stronger bilateral activation of the precentral gyrus.

#### 3.2.2. Within-Group Comparison

**Figure 6:**
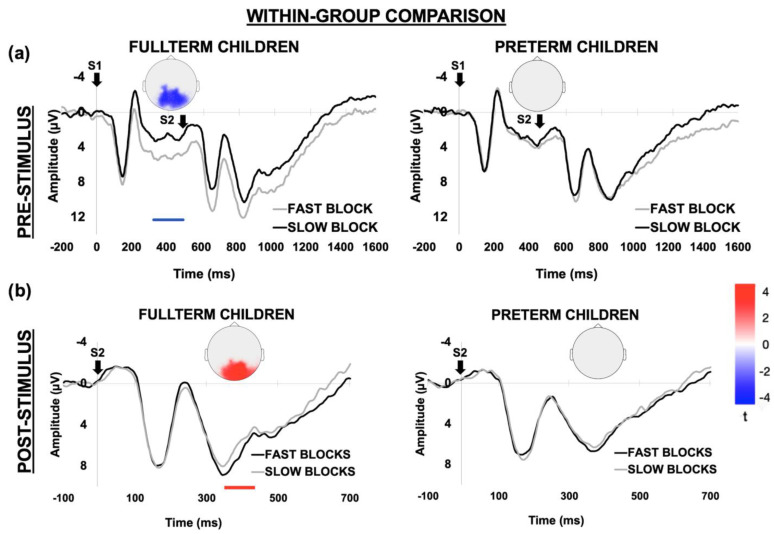
Within-group ERP comparison. The upper panel (**a**) represents the statistically significant electrodes exceeding the critical t-score threshold for statistical significance over the pre-stimulus window (stimulus expectancy/response preparation). The negative cluster indicates that ERP mean amplitude is significantly larger (i.e., more negative) in the fast than slow blocks over posterior electrodes in the FT group. This effect extended over a 300 ms window before S2 onset and was expressed by a CNV modulation (left panel). However, no significant block-dependent modulations were found in the PT group. The ERP waveforms below the scalp maps show the time series derived by averaging the electrodes included in the negative cluster. For visual comparison purposes, the time series derived from the same cluster of electrodes was shown for the PT group (right panel). The S1 at 0 ms indicates the ERP time locking, while S2 indicates the onset of the imperative stimulus. The lower panel (**b**) represents the same analysis performed over the post-stimulus window (stimulus processing/response implementation). The negative cluster indicates that ERP mean amplitude is significantly larger (i.e., more positive) over posterior electrodes in the fast than slow blocks for the FT but not PT group. The ERP waveforms below the scalp maps show the time series derived by averaging the electrodes included in the positive cluster. These clearly showed a larger P3 amplitude in fast than slow blocks only for FT (left panel). For visual comparison purposes, the time series derived from the same cluster of electrodes was shown also for the PT group (right panel). The S2 at 0 ms indicates the ERP time locking. For all comparisons the colored horizontal bars represent the timing of significant ERP effects.

To investigate the neural correlates of ACC, we run two second-level permutative analyses which targeted ERP modulations as a function of global stimulus predictability. In particular, we compared the ERP activity elicited in fast vs. slow blocks, separately by group and only for short-SOA trials. In this way we were able to investigate whether the block-level group differences in speed adjustment were supported by a different recruitment of neural resources. The fine temporal resolution of ERPs allowed us to unveil the temporal dynamics of ACC, targeting both pre- and post-stimulus windows as two computational stages reflecting stimulus expectancy and response implementation, respectively. According to H2b, and in line with behavioral findings, we expected to find a larger neural modulation in FT than PT children. As shown in Figure 6a, FT children showed significantly higher pre-stimulus ERP activity in fast than slow blocks, which was expressed by a more negative CNV (*p* = 0.034, cluster statistic (c) = −64, cluster size (s) = 56). This effect involved a large cluster of posterior electrodes, covering occipito-parietal sites. By contrast, no significant ERP modulations induced by global stimulus predictability were observed in the PT group. Likewise, in the post-stimulus window (Figure 6b) FT children allocated larger neural activity following S2 when this occurred in fast as compared to slow blocks (*p* = 0.028, cluster statistic (c) = 60, cluster size (s) = 50). This effect was expressed by a larger P3 over posterior electrodes. By contrast, no significant post-stimulus ERP modulations were found in PT children. 

**Figure 7:**
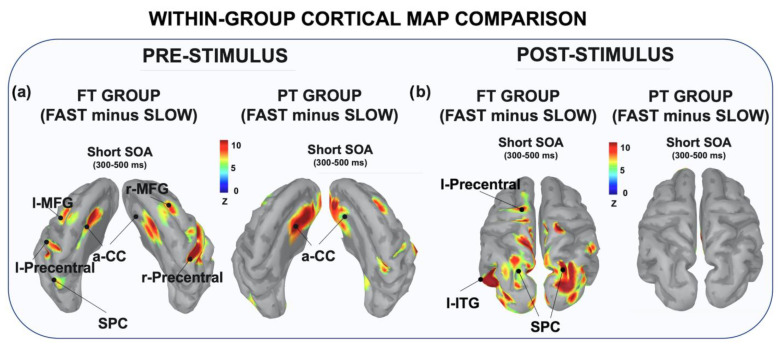
Within-group differential cortical map reconstruction. The pictures display the differential (fast minus slow blocks) cortical map reconstruction relative to each significant ERP effect for pre-stimulus (**a**) and post-stimulus (**b**) temporal windows. Data were adjusted using a threshold of 50% of the maximum amplitude and a size of at least 10 vertices and they were reported on a template cortex smoothed at 40%.

The visual inspection of the source map reconstruction revealed high cortical activity in FT than PT children during the fast blocks, over both pre- and post-stimulus time windows. More specifically, as shown in Figure 7, during the pre-stimulus window the FT exhibited larger neural activity when engaged in fast vs. slow blocks over a distributed pattern of cortical areas. These included the precentral gyrus, the MFG and the aCC bilaterally together with the l-SPC. By contrast, the PT group showed a reduced cortical pattern, mainly involving the bilateral aCC and the r-MFG. 

When looking at the post-stimulus time window we still observed a much larger activity in FTs spreading over posterior (occipital, parietal and temporal) and central (left precentral gyrus) areas.

## 4. Discussion

In this study we investigated ACC in preterm children, addressing the specific question of whether they succeed in extracting and using local and global S-R temporal regularities to generate, implement and update temporal prediction and, consequently, adapt CC for optimal action preparation. We used the DTP, a task originally created by our laboratory [41] in which S-R temporal contingency is manipulated unbeknownst to participants to generate either local (trial-wise expectancy) or global (block-wise expectancy bias) stimulus prediction. 

Our findings show that preterm (PT) children are overall less efficient in using CC than full-term (FT) children both at the behavioral and neurofunctional level, confirming our first hypothesis (H1). Indeed, they showed overall slower and more premature responses in the DTP task as compared to FT peers. Moreover, we also documented a reduced amplitude of both pre-stimulus (CNV) and post-stimulus (N1–N2) electrophysiological activity in PTs, clearly indicating that premature birth negatively impacts both response preparation and stimulus processing. These findings are in line with previous studies consistently reported slow processing speed [76,77,78], inhibitory control [79,80,81] and suboptimal neural recruitment underlying attentional control and motor resource allocation during response preparation [76,77]. This altered neuropsychological pattern has been related to the presence of both gray and white matter abnormalities typically observed as a consequence of premature birth [82], potentially implying a disruption of cortical and subcortical functional connectivity. Even though our data do not allow direct address of this hypothesis, the differential cortical activation observed during both response preparation and stimulus processing is in line with this interpretation. Indeed, we report that PT children show lower recruitment of frontal (precentral gyrus), prefrontal (anterior cingulate cortex, middle frontal gyrus) and parietal (superior parietal cortex) areas. More precisely, as we previously suggested [56], the engagement of posterior areas in S1–S2 tasks may probably reflect the allocation of attentional resources on S1 processing by itself. Differently, the frontal and prefrontal areas have been traditionally associated with action preparation/control (anterior cingulate cortex) and execution (precentral gyrus) before S2. This pattern has been consistently reported in previous studies employing experimental tasks inducing proactive control on the basis of both explicit [24,25,56] and implicit [56,83] temporal prediction. From a systemic viewpoint, neuroimaging investigations further demonstrated that these areas are highly functionally connected each-other to form distinct circuits underlying CC, including the cingulo-opercular and the fronto-parietal networks. Moreover, recent studies showed that the subtle white and gray matter abnormalities resulting from preterm birth may underlie the documented altered functional connectivity in both these networks [84,84,85]. However, further research is needed to better understand the relationship between altered structural and functional brain connectivity and ACC. Interestingly, previous evidence of suboptimal cognitive flexibility in PT is further corroborated by our results also at the behavioral level showing that PT children committed more perseverative errors in the WCST, as evidence of suboptimal cognitive flexibility in this population [1,8]. Taken together, the between-group comparison supported H1 both at the neural and behavioral levels, clearly showing a less efficient CC in children born prematurely, with regard to speed processing, response inhibition, response preparation and cognitive flexibility. It is noteworthy to consider that our sample was undiagnosed and did not present academic skill problems. Hence, the CC weakness here documented could represent a sub-clinical endophenotypic marker characterizing the neurocognitive profile of children born preterm, even when they do not meet the criteria for clinical diagnosis.

Noteworthy, despite slower response speed and lower response preparation, premature children succeeded in adapting their performance as a function of implicit stimulus temporal predictability. However, as expected (H2), we found that the efficiency of this mechanism depended on the type of implicit knowledge they used. More specifically, both groups were able to speed up their response times (although these differed in absolute terms) as a function of local stimulus predictability, showing longer RTs for shorter SOA trials and shorter RTs for longer SOA ones. This SOA-related behavioral advantage can be explained by the increase in the conditional probability of stimulus presentation as a function of time (hazard rate) [51] (p. 20), [53,57]. Previous studies demonstrated that the local prediction effect is already present from early childhood in typical [57,60], atypical (Down syndrome; [42,86]), neurological [43] and at-risk [45] developmental population. Here we further extend previous evidence that ACC can be shaped by local temporal prediction also in PT children. It has been proposed that hazard-related local prediction is an automatic mechanism, which does not require effortful control. As such, it is not surprising that it emerges early across typical development, is preserved in atypical development and does not show sensitivity in children showing neurodevelopmental vulnerability, as PT children do.

However, differently from local prediction, when looking at the effect of global stimulus predictability we found a different pattern for FT and PT groups. Indeed, while all children adapted their response speed to task changes, showing faster RTs when the stimulus presentation rate increased (i.e., fast blocks), the mean behavioral advantage (Δ global) obtained was significantly larger in PT than FT children. At a first sight this could appear controversial, as one should expect that larger behavioral gain may represent a better CC adaptation. However, in the context of the DTP task a more efficient behavioral adaptation is reflected by a smaller relative speed modulation. Previous studies from our laboratory indeed clearly showed (1) smaller Δ values with increasing age in typically developing children, suggesting an age-dependent refinement of the capacity to flexibly adapt behaviour to complex statistical patterns [41,87] and (2) larger delta values in children with neurodevelopmental risk related to neurological disorders [43]. Hence, in line with previous studies, here we confirm that the DTP task is able to sensitively discriminate PT from FT not only with regard to the overall CC performance (response speed, inhibition and flexibility) but also in terms of response speed adaptation to contextual factors (implicit global prediction), proving a reliable tool for measuring ACC. As an additional noteworthy finding, the mean speed adaptation in the DTP (Δ global) was positively related to WCST performance, with higher values predicting higher percentages of perseverative responses. This effect was independent from the child’s age and suggests that explicit CC (top-down cognitive flexibility upon explicit instruction) is associated with implicit CC (behavioral adaptation based on implicit bottom-up information). This further suggests that CC can be conceived as a transversal, endophenotypic individual characteristic that operates regardless of either the representational level of the information to be controlled or one’s awareness of having to use control. Whether explicit and implicit CC represent two sides of the same coin remains to be understood, as recent studies using other tasks showed a developmental dissociation between them [39]. 

Besides any theoretical implication, from an educational and clinical perspective, these findings suggest the potential benefit of helping PT children with low ACC, providing them some hints to strategically and deliberately pay attention to contextual environmental changes, as they may struggle to automatically exploit the environmental changes when controlling behaviour, thoughts and emotions.

Furthermore, in line with hypothesis H3, we found that the differences in speed adaptation as a function of global changes are supported by a different within-group neural modulation. Specifically, the request of increased CC during fast blocks induced a larger recruitment of neural activity in FT but not PT children. At the scalp-level, this was observed by a larger electrophysiological activity during fast than slow blocks. Indeed, we clearly observed that the RT speeding up induced by the increased stimulus rate presentation in fast blocks was supported by a larger anticipatory CNV. Noteworthy, the within-group CNV modulation was expressed over posterior scalp sites. This posterior modulation was already observed in a previous study by our laboratory (Mento and Vallesi, 2016). In this study we presented healthy children with explicit cues providing either predictive or neutral information about the timing of imperative stimuli. When allowed to use proactive CC based on predictive cues children were overall faster and showed a larger posterior CNV amplitude as compared to when they could only use reactive CC (neutral cues). Here we replicate this finding with a different task exclusively tapping on implicit information to adapt CC from reactive to proactive mode.

At the source-level, we found that the cortical areas underlying response preparation and stimulus processing exhibited larger activity in fast blocks. Interestingly, during response preparation the activity of prefrontal (MFG) and frontal (precentral gyrus) areas extended bilaterally over both hemispheres, suggesting that ACC operates by engaging a wider fronto-parietal network. Remarkably, in PT we only found a bilateral involvement of the anterior cingulate cortex. A possible explanation for this different cortical pattern is that PT children are actually able to instantiate CC as a function of global prediction, recruiting medial prefrontal cortex to sustain the request for an enhanced effort. However, they do not succeed in proactively translating this enhanced cognitive effort into motor control, failing to efficiently adapt response speed up to task demand in a prompt way. These findings complemented behavioral and electrophysiological evidence by providing a possible neurofunctional explanation about the less efficient global response adjustments observed in the PT group. 

Taken together, the findings we report in the current study provided new insights into the mental functioning of preterm children. On the one hand, we corroborated previous findings showing a poor CC when PT children are required to produce speeded responses to warned visual stimuli and to flexibly adapt their response choices to abstract rule changes. On the other hand, we extended previous knowledge by providing novel evidence of poor ability of PTs to flexibly adapt CC as a function of contextual environmental information, namely, implicit global changes in stimulus prediction. The use of HD-EEG further allowed us to depict the spatiotemporal neuro-dynamics underlying between (CC) and within-group (ACC) effects, advancing some tentative hypotheses about the neurofunctional locus of the different behavioral patterns observed in prematurely born children. It is nevertheless crucial to highlight that, as far as we know, this is the first study purposely using the DTP task to investigate ACC in this population, and that the inference we can draw from brain source reconstruction must be considered carefully due to the sub-optimal spatial resolution of EEG. Hence, further investigation using different tasks and high-spatial resolution neuroimaging tools will be needed for a better understanding of ACC functioning in preterm children.

## Figures and Tables

**Table 1:** Demographic characteristics of the participants. PT = Preterm; FT = Full-term.

	PT	FT
N Tot (% Males)	21 (52%)	21 (42%)
Age in Years M (SD)	8.42 (1.3)	8.19 (1.4)
Gestational Age in Days M (SD, Range)	213 (20.9; range 168–252)	>270
Birth Weight in Grams M (SD)	1448 (623)	>2500

**Table 2:** Accuracy Mean and standard deviation (in parentheses) measures of accuracy (percentage of premature responses) for preterm (PT) and full-term (FT) children for each experimental condition.

BLOCK	FAST			UNIFORM			SLOW		
SOA	Short	Medium	Long	Short	Medium	Long	Short	Medium	Long
PT	94.3 (6.1)	93.1 (7.1)	93.8 (9.4)	92.3 (5.6)	95.7 (4.6)	93.8 (5.3)	92.2 (7.4)	93.7 (7.1)	92.9 (6.4)
FT	97.7 (2.2)	96.8 (3.7)	91.8 (5.7)	97.6 (4)	96.9 (4.1)	96.7 (4.4)	98.6 (4.9)	96.8 (4)	97 (2.9)

**Table 3:** Response speed. Mean and standard deviation (in parentheses) measures of response speed (reaction times) for preterm (PT) and full-term (FT) children for each experimental condition.

BLOCK	FAST			UNIFORM			SLOW		
SOA	Short	Medium	Long	Short	Medium	Long	Short	Medium	Long
PT	477 (84)	418 (88)	402 (90)	508 (88)	441 (99)	402 (70)	521 (86)	444 (92)	420 (79)
FT	507 (76)	444 (80)	438 (86)	570 (68)	492 (86)	457 (68)	602 (92)	510 (86)	475 (86)

## Data Availability

Behavioral and EEG data are freely accessible on Open Science Framework (Available online: https://osf.io/mqzet/ (accessed on 21 July 2022).
